# Quarantining arriving travelers in the era of COVID-19: balancing the risk and benefits a learning experience from Bahrain

**DOI:** 10.1186/s40794-020-00128-w

**Published:** 2021-01-12

**Authors:** Abdulkarim Abdulrahman, Manaf AlSabbagh, Abdulla AlAwadhi, Jaffar A. Al-Tawfiq, Ali A. Rabaan, Stephen Atkin, Manaf AlQahtani

**Affiliations:** 1 National Taskforce for Combating the Corona Virus, Riffa, Bahrain; 2 Mohammed bin Khalifa Cardiac Centre, Riffa, Bahrain; 3 Planning and Economic Studies Directorate, Ministry of Finance and National Economy, Manama, Bahrain; 4 Department of Medicine, Bahrain Defense Force Hospital, Riffa, Bahrain; 5 Infectious Disease Unit, Specialty Internal Medicine, Johns Hopkins Aramco Healthcare, Dhahran, Saudi Arabia; 6 Department of Medicine, Indiana University School of Medicine, Indianapolis, IN USA; 7 Department of Medicine, Johns Hopkins University School of Medicine, Baltimore, MD USA; 8 Royal College of Surgeons in Ireland-Bahrain, Muharraq, Bahrain; 9 Internal Medicine, Infectious Diseases and Clinical Microbiology, Bahrain Defense Force Hospital, Riffa, Bahrain

**Keywords:** Travelers, Quarantine, COVID19, SARS-CoV2, Public health

## Abstract

The quarantine period imposed to travelers in many countries due to COVID19 is a major obstacle for any traveler. Lifting the quarantine period could lead to significant improvement in people’s quality of life and any country’s economy. Bahrain have used two quarantine models from arriving passengers. We report data about the incidence of COVID19 on arriving passengers at Bahrain International airport. Infection rates were reported on arrival, during quarantine and after leaving quarantine. Results showed that travelers had low incidence of COVID19 on arriving and during the quarantine period, while becoming at higher risk after leaving quarantine. We concluded that quarantine requirement maybe lifted for arriving travelers. Testing upon arrival with implementation of the public health preventative measures can minimize the risk of transmission.

## Background

The outbreak of severe acute respiratory syndrome coronavirus 2 (SARS-CoV-2) infections have infected more than 65 million people worldwide and Bahrain has witnessed over 87,000 cases to date [1]. This pandemic had its implications on all aspects of life, and it especially affected many countries economically. Different countries instated quarantine measures for international arrivals. A 14 day quarantine with testing upon exit has been a common practice [2].

The quarantine period is an obstacle for travelers. There is potential improvement in people’s quality of life (QOL) and to the country’s economy should this quarantine period be lifted. Quarantine hinders people from attending their work and business in timely manner. There are issues that require urgency such as, diplomatic missions and urgent business dealings, which could be delayed and canceled due quarantine. Implementing quarantine incur a significant cost to the health authorities, as there is a need to test, track and follow up. This can prove to be a financial and a resources burden. Quarantine also have a negative psychological effects. Stemming from people lack ability of managing quarantine challenges; Such as, social isolation, loss of freedom, and uncertainty over disease status [3].

Maldives, Turkey, UAE, Mexico and Brazil have removed the quarantine period for visitors. Other countries have stratified arrivals based on the risk of the country of origin. This has been a common measures in European countries like Austria, Slovakia, Switzerland and Netherlands.

The quarantine measures may not be necessary. In this manuscript we discuss Bahrain’s data and experience about quarantining of international travelers.

## Main text

The quarantine measures were especially important early during the pandemic, when the majority of cases in Bahrain were travelers. Between 24 February and 31 March, travelers made up 60% of all cases in Bahrain, with the local cases being contacts of those travelers. As local transmission within the country grew, local cases became the majority of cases. In April and May, local transmission cases made up more than 95% of all cases in Bahrain. This was due to increased local transmission and decreased in traveling. There were 8477 cases due to local transmission in May compared to 2349 cases in April.

We retrospectively examined the data of all arrivals at Bahrain International Airport between 15th May to 30th June 2020. There were 10,784 passengers arriving and all were tested. 335 (3.1%) tested positive upon arrival. All other travelers entered quarantine for 14 days after testing negative. Only 63 passengers (0.6%) who entered quarantine tested positive. Furthermore, 276 (2.6%) of the arrivals who were negative after completion of the quarantine period, got infected by coming in contact to a positive case in Bahrain.

Several studies report that most infected individuals are no longer contagious 10 days after symptoms development [4, 5]. The incubation period for SARS-CoV-2 was thought to have a median time of 4–5 days from exposure to symptoms onset [6]. 97.5% of infected people who develop symptoms would do so within 11.5 days of SARS-CoV-2 infection [6]. Moreover, it was observed that the time from exposure to onset of infectiousness was shorter than the incubation period estimated [6]. A Chinese study suggested that infectiousness starts 2.3 days prior to symptom onset [7]. Hence, the Bahraini authorities concluded that a PCR test through a NP sample at day 10 would be sufficient to detect at least 97.5% of all infected individuals. Moreover, the authorities also issued new guidelines for international passengers where quarantine rules were imposed for 10 days upon arrival after their first RT-PCR test.

Based on this policy, another analysis of the data was conducted. Between 1st July & 16th August, all 15,070 arrivals at Bahrain International Airport were tested. 178 (1.2%) tested positive upon arrival. 28 (0.2%) passengers tested positive during the 10 days quarantine period. 312 (2.1%) tested positive while in Bahrain due to local transmission.

Conversely, under these two models of quarantine protocols, only few cases turned positive during quarantine (Table 1). The percentage of arrivals diagnosed with SARS-CoV-2 infection after leaving quarantine did not increase and remained low. Thus, quarantine rules were withdrawn, and the testing was continued for all passengers upon arrival.

**Table 1:** Summary of the returning travelers in quarantine with percent of positive SARS-CoV-2 test

Study Period	Days of Quarantine	Number of travelers	SARS-CoV-2 positive on arrivals	SARS-CoV-2 positive during the quarantine period	SARS-CoV-2 positive due to local transmission
May 15–June 30, 2020	14	10,784	335 (3.1%)	63 (0.6%)	276 (2.6%)
July 1–August 16, 2020	10	15,070	178 (1.2%)	28 (0.2%)	312 (2.1%)

With the 14 days quarantine, 2.6% were infected after the quarantine period and 0.6% within the quarantine period. With the 10-day quarantine, 2.1% were infected after the quarantine period and 0.2% within the quarantine period. There was no difference in these two quarantine strategies employed for the travelers

With community transmission rising in the country, travel restriction and compulsory quarantine alone cannot control the outbreak. Preventive measures such as self-isolating if symptomatic, social distancing, wearing a face mask, washing hands and proper hygiene maintenance is more crucial than quarantining travelers. These measures, in addition to testing on arrival, can be sufficient to prevent the transmission of an infected case traveling into the country and can improve the country’s economy and the QOL of the arrivals. This is especially important as reopening measures have been planned and starting.

## Conclusion

The results from our study showed that only a minority of travelers were infected with SARS-CoV-2. This highlights that quarantine for travelers may not be necessary. However, testing upon arrival with implementation of the public health preventative measures can minimize the risk of viral transmission.

## Data Availability

The datasets used and/or analyzed during the current study are available from the corresponding author on reasonable request.

## Abbreviations

SARS-CoV-2: Severe acute respiratory syndrome coronavirus 2
COVID-19: Corona Virus Disease of 2019
RT-PCR: Real time polymerase chain reaction
NP: Nasopharyngeal
QOL: Quality of life
